# A Novel Adversarial Deep Learning Method for Substation Defect Image Generation

**DOI:** 10.3390/s24144512

**Published:** 2024-07-12

**Authors:** Na Zhang, Gang Yang, Fan Hu, Hua Yu, Jingjing Fan, Siqing Xu

**Affiliations:** State Grid Shanxi Electric Power Research Institute, Taiyuan 030001, China

**Keywords:** generation of defect images for substation equipment, GAN, local region defect generation, joint discriminator for overall image and defect image

## Abstract

The presence of defects in substation equipment is a major factor affecting the safety of power transmission. Therefore, timely and accurate detection of these defects is crucial. As intelligent inspection robots advance, using mainstream object detection models to diagnose surface defects in substation equipment has become a focal point of current research. However, the lack of defect image data is one of the main factors affecting the accuracy of supervised deep learning-based defect detection models. To address the issue of insufficient training data for defect images with complex backgrounds, such as rust and surface oil leakage in substation equipment, which leads to the poor performance of detection models, this paper proposes a novel adversarial deep learning model for substation defect image generation: the Abnormal Defect Detection Generative Adversarial Network (ADD-GAN). Unlike existing generative adversarial networks, this model generates defect images based on effectively segmented local areas of substation equipment images, avoiding image distortion caused by global style changes. Additionally, the model uses a joint discriminator for both overall images and defect images to address the issue of low attention to local defect areas, thereby reducing the loss of image features. This approach enhances the overall quality of generated images as well as locally generated defect images, ultimately improving image realism. Experimental results demonstrate that the YOLOV7 object detection model trained on the dataset generated using the ADD-GAN method achieves a mean average precision (mAP) of 81.5% on the test dataset, and outperforms other image data augmentation and generation methods. This confirms that the ADD-GAN method can generate a high-fidelity image dataset of substation equipment defects.

## 1. Introduction

### 1.1. Background

The substation achieves the transmission of electricity from power plants to end users through the conversion of high and low voltages, and is an indispensable part of the power system. Monitoring the operational status of substations is crucial for the stable operation of the power grid. In the past, the detection of defects in substations, such as rust, oil leakage, damaged dials, and fractured insulators, was typically carried out through manual inspection, which was time-consuming and labor-intensive [1]. With the development of remote observation technologies for substations, images captured by cameras can be used to remotely monitor the equipment’s operational status. However, even the assessment of defects in equipment images is carried out manually. During the prolonged image screening process, manual judgment can lead to eye fatigue, resulting in potential errors, omissions, and subjective biases. With the continuous improvement in smart grid construction, it is imperative to implement unmanned and intelligent monitoring for substations [2]. Automatically detecting equipment images through image processing methods not only avoids errors caused by subjective factors but also enhances the detection efficiency and level of intelligence in substations [3].

### 1.2. Motivation and Related Works

In recent years, with the construction of smart substations, the application of unmanned visual monitoring systems, ground inspection robots, and aerial inspection drones has gradually become widespread [4,5]. An increasing number of surface defect detection methods for substation equipment based on deep learning have been applied in practical inspection tasks [6,7,8,9]. These methods are often improvements upon supervised object detection models like YOLO, effectively enhancing the detection performance of surface defects in substation instruments. This provides favorable conditions for the intelligent monitoring of unmanned substations.

The surface defect detection methods for substation equipment based on supervised deep learning mentioned above require a sufficient amount of training data to support their accuracy. However, the construction of surface defect datasets for substation equipment faces the following challenges: (1) In the process of routine inspection and monitoring data collection, normal images of substation equipment overwhelmingly outnumber defective images [10]. This imposes a significant workload on the manual screening and classification of defective images. (2) There is a considerable variation in manual expertise, leading to significant differences in the collection, classification, and annotation quality of defective images, making it difficult to ensure the quality of subsequent dataset construction. (3) Current dataset augmentation primarily relies on techniques such as image rotation, stretching, and brightness transformation, with limited utilization of image super-pixel features. This results in high redundancy of the dataset and poor improvement in the recognition and detection models.

To address the aforementioned issues, many studies have applied data augmentation methods to various fields such as insulator fault detection in diverse aerial images [11], surface defect detection in industrial components [12], etc. These studies have conducted research on image generation methods based on superpixel features. Through generative models such as auto-encoders (AEs) [13] and generative adversarial networks (GANs) [14], they generate new images with complex backgrounds and diverse features. This process helps in constructing training datasets with higher richness, thereby further enhancing the performance of defect classification and detection models. Di Maggio et al. [15] propose a novel method using CycleGAN [16] to generate data for damaged industrial machinery. By training the generative model, it transforms wavelet images simulating vibration signals into real data from mechanically damaged bearings. To enhance the efficiency and lower the expense of detecting cracks in large-scale concrete structures, Liu et al. [17] generated a large number of artificial samples from existing concrete crack images by DCGAN [18], and the artificial samples were balanced and feature-rich. Zhuang et al. [19] employed a generative adversarial network based on Defect-GAN [20] to augment and expand a small sample image dataset of plastic label defects. This method, by simulating the process of defect generation and defect image reconstruction, can synthesize defect samples with high fidelity and diversity. To address the issue of limited samples of abnormal defect images on the surface of trains, Liu et al. [21] proposed an Anomaly-GAN based on mask pool, abnormal aware loss, and local versus global discriminators. This method preserves the global and local authenticity of the generated defect images.

Although the aforementioned image enhancement methods based on adversarial deep learning generate new industrial defect images, there are still several drawbacks when applied to the generation of substation equipment defect images: (1) Substation equipment images are captured in real-world environments with complex backgrounds. The processing steps in the mentioned methods intentionally weaken complex backgrounds, presenting a limitation in existing adversarial deep learning methods, as they can only transform the overall image style and not specifically the style within the region of interest. (2) Substation equipment defect images exhibit complex features such as rust and oil leakage, which not only change the color of the equipment surface but also include rich texture features. The GAN-based methods mentioned above primarily focus on simple defects like scratches and ink dots, which are easier to generate compared to the more challenging and diverse defects found in substation equipment, leading to higher success rates. (3) The quality of generated images needs further improvement. Existing GAN methods often encounter mode collapse during the training process [22,23], blurring the details of the generated defects. Furthermore, if the generated images lack realism and stability, the relationship between the generated defects and the overall image becomes conspicuously unnatural.

### 1.3. The Key Contributions and Innovations

To address the aforementioned challenges, we proposes an abnormal defect detection generative adversarial network (ADD-GAN) model tailored for typical substation defects like rust and oil leakage. The key contributions and innovations are summarized as follows: (1) Effective Region Extraction: The ADD-GAN model can extract effective generation regions in the complex background of original images. By transforming regions of normal equipment images into images with defects such as rust and oil leakage, it resolves the issue of an insufficient number of original defect images. (2) Local Region Defect Generation: Introducing a local region defect generation network, ADD-GAN generates defects on substation equipment images based on the extraction of effective generation regions. This approach avoids a decrease in image realism caused by transforming features across the entire image. (3) Joint Discriminator for Overall Image and Defect Image: ADD-GAN introduces a joint discriminator for overall image and defect image, enhancing the quality of generated defect images. This method focuses on generating defect images in specific regions of substation equipment while ensuring the overall quality of the generated images, thus avoiding distortion caused by changes in the global image style. (4) Loss Function Design: The ADD-GAN model is designed with global perceptual adversarial loss, local defect perceptual loss, and cycle consistency loss. This ensures the realism of the overall image and the rationality of locally generated defect images, and prevents mode collapse.

The following content is structured as follows: Section 2 introduces the composition of the original dataset; Section 3 provides a detailed explanation of the algorithmic principles of the ADD-GAN model; Section 4 discusses related experimental work; and the final section presents a conclusion.

## 2. Dataset Preparation

**Figure 1:**
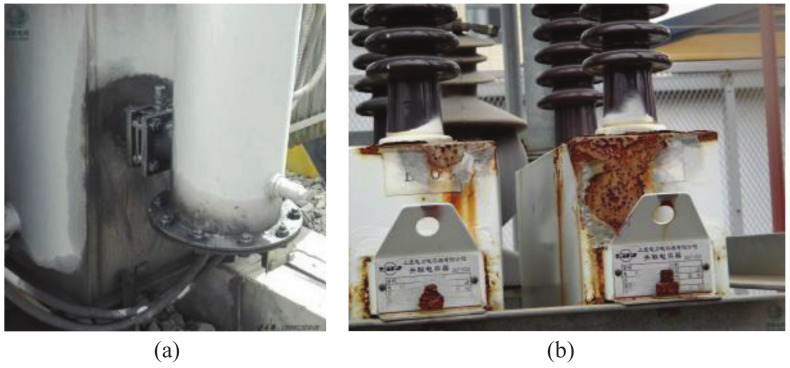
Typical appearance defects of substation equipment: (**a**) Equipment surface leakage of oil. (**b**) Rust.

Transformer and other equipment are crucial components for the normal operation of power systems. Incidents such as surface oil leakage and rust can adversely affect the proper functioning of these devices, posing potential safety hazards when severe. The visual manifestations of surface rust and oil leakage are depicted in Figure 1, making them essential inspection targets during both manual and automated inspections. However, during inspections, obtaining normal images of substation equipment is more common, with a relatively small overall number of defective images and an imbalance in the number of images between classes. Therefore, image generation is carried out for the above two types of defects.

From 1 January 2023 to 5 March 2024, we collected 700 images of surface defects from substation equipment in Taiyuan City, Shanxi Province, and Jinan City, Shandong Province, China. This dataset includes 353 images of surface oil leakage and 347 images of metal rust. Additionally, we gathered 1200 images of normal equipment. Notably, collecting images of normal equipment was significantly less challenging compared to capturing images of the two types of defects.

## 3. Materials and Methods

This section introduces three concepts and their principles: local defect image augmentation, the ADD-GAN model, and the objective loss function. These are employed for generating corresponding equipment defect images.

### 3.1. Local Defect Image Augmentation

To fulfill the data number demands of deep learning models, this section introduces a method for local defect image augmentation to increase the diversity of abnormal samples.

Elements in the defect image dataset have two main sources. The first source involves manually segmenting local defect images based on prior knowledge from the collected original dataset of equipment defect images. The second source includes local defect images based on expert experience drawn by experts using tools. The two types of images, namely the defect images created by experts and the generated defect images, show variations in size, form, and spatial arrangement. To ensure the authenticity of the expert-created defect images, we follow specific guidelines during the drawing process that correspond to real-world scenarios, as shown in Table 1. After generating new defect images, an expert evaluation approach is used to assess the accuracy and reasonableness of the expert-created defects. Ten experts evaluate the plausibility of the new images, and the resulting score for each image is calculated as the average of the expert ratings. Subsequently, the defect images drawn by experts that fall within the top 15% are retained.

In order to further enhance the quantity and variety of new samples, we employ random repositioning, rotation, and scaling techniques for defect images from both groups. Moreover, various defect components are randomly merged together to introduce a larger number of defects within a single image. This approach effectively reduces the manual drawing work burden. By combining these artificially generated defects with real collected images, we form the training dataset for subsequent GAN model training.

### 3.2. ADD-GAN Algorithm

#### 3.2.1. Algorithm Principle of GAN Model

Generative Adversarial Network (GAN) is a widely used image generation model. It learns from a set of data and generates similar data. The GAN model consists of two parts: the generative model and the discriminative model. The generator captures the distribution of sample data and uses noise *z* following a certain distribution (uniform distribution, Gaussian distribution, etc.) to generate a sample resembling real training data. The goal of the generator is to produce samples that become increasingly similar to real samples.

The discriminator is a binary classifier used to estimate the probability that a sample comes from the training data rather than the generated data. If the sample comes from real training data, the discriminator outputs a high probability; otherwise, it outputs a low probability. During the training process, the GAN model fixes one of the generator *G* or discriminator *D*, updates the weights of the other, and iterates alternately. The formula is as follows:

(1) $$\underset{G}{min}\underset{D}{max}V\left(D,G\right)=E_{x∼P_{data}\left(x\right)}\left[lgD\left(x\right)\right]+E_{z∼P_{data}\left(z\right)}\left[lg\left(1-D\left(G\left(z\right)\right)\right)\right],$$

where $P_{data}\left(x\right)$ represents the distribution of real sample *x*, $P_{data}\left(z\right)$ shows the distribution of random sample *z*, and V(D,G) is the difference between *D* and *G*. The purpose of the *G* is to minimize V(D,G) as much as possible while keeping *D* fixed, and the purpose of *D* is to maximize V(D,G) while keeping *G* fixed. During the training process, both *G* and *D* continuously optimize their networks, forming a competitive objective until both parties reach a Nash equilibrium. At this point, *G* can generate samples identical to the real data distribution, and *D* achieves an accuracy of 50% in discriminating generated samples. The final generative model is then used to generate the required data.

**Figure 2:**
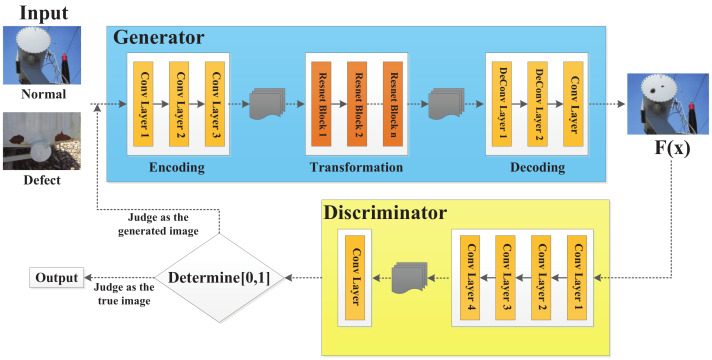
Structure diagram of ADD-GAN model.

The majority of existing GAN models generate defect images with unclear local details and limited image diversity. However, supervised defect detection models necessitate training with defect images characterized by a high degree of diversity and distinct details. To overcome this challenge, this paper introduces the ADD-GAN model, depicted in Figure 2. ADD-GAN operates on the principle of transforming one class of images into another. It involves two sample spaces, denoted as *X* and *Y*, representing the sets of normal equipment image samples and images with oil leakage or rust, respectively. The objective is to transform samples from space *X* to space *Y*. Therefore, the goal of ADD-GAN is to learn the mapping function *G* from *X* to *Y*. *G* corresponds to the generator in the ADD-GAN network model, capable of generating an image F(x) in space *Y* from an image *x* in space *X*. For the generated image F(x), the discriminator $D_{Y}$ in ADD-GAN is employed to determine whether it is a real image, forming an adversarial neural network. The ADD-GAN model facilitates the bidirectional transformation between normal and defective images. In this study, the focus is primarily on converting normal images of substation equipment into defective images.

#### 3.2.2. Local Region Defect Generation Network

At present, image generation methods using GAN networks often prioritize texture transformation for the overall image while neglecting the fine-grained defect features in small regions. In other words, not only are defective images of the surface of substation equipment generated but the overall style of the entire image also undergoes changes. To address these issues, this paper introduces a Local Region Defect Generation Network during the image generation stage. Through image segmentation, it initially isolates the region for defect generation. Subsequently, local style transformation and defect generation are applied to this segmented region. This modification enhances the performance of ADD-GAN in the generation of semantic features for small region defects.

**Figure 3:**
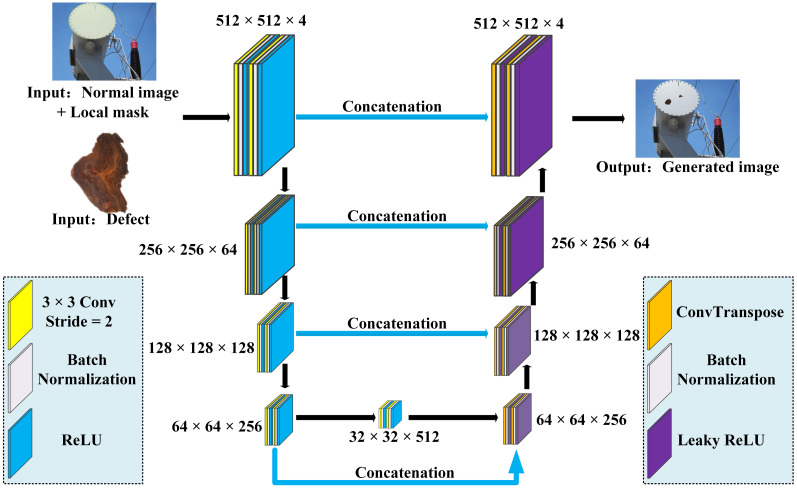
Generator based on U-Net structure.

In this paper, an encoder–decoder network based on the U-shaped architecture (U-Net) [24] is designed to constitute the local region defect generator model, as shown in Figure 3. U-Net was initially employed in the field of medical image processing for segmenting medical images. Improved from a fully convolutional neural network, U-Net is named after its shape resembling the letter “U”. It consists of an encoding path and a decoding path. The encoding path is a typical convolutional neural network, comprising a repetitive functional structure. Each structure employs the combination function ConV-BN-ReLU-ConV-BN-ReLU for the processing of feature maps along the path, where ConV stands for convolution, BN for batch normalization, and ReLU for rectified linear units. After each downsampling, the number of feature channels is doubled. In the decoding path, each step begins with the use of the transposed convolution (ConvTranspose) operation, where the feature map size is doubled and the number of feature channels is halved after each transposed convolution. Following ConvTranspose, the result is concatenated with the corresponding-sized feature map from the encoding path and propagated backward. This encoding and decoding process, along with feature concatenation, allows for better fusion of shallow pixel position features with deep pixel category features, improving the utilization of image features and contributing to enhanced image generation. Therefore, this study employs the U-Net architecture instead of the fully convolutional neural network architecture, and designs the local region defect generator model.

As shown in Figure 3, the input to the generator includes not only the normal device image but also one-dimensional mask information. This information is pre-set and can be obtained through manual annotation or generated automatically using deep learning-based image segmentation methods. During the image generation process, the original image and mask information are input together into the generator model. The generator utilizes the mask information to first segment the local area of the device in the original image where defects need to be generated. Subsequently, the required defects are generated in the segmented area. After completing the defect generation, the output result replaces the mask region on the original image, completing the generation of the defect image. This approach not only retains the advantages of GAN model style transfer but also restricts the range of image style transformation to the desired local area, significantly enhancing the authenticity of generated defect images.

#### 3.2.3. Joint Discriminator for Overall Image and Defect Image

**Figure 4:**
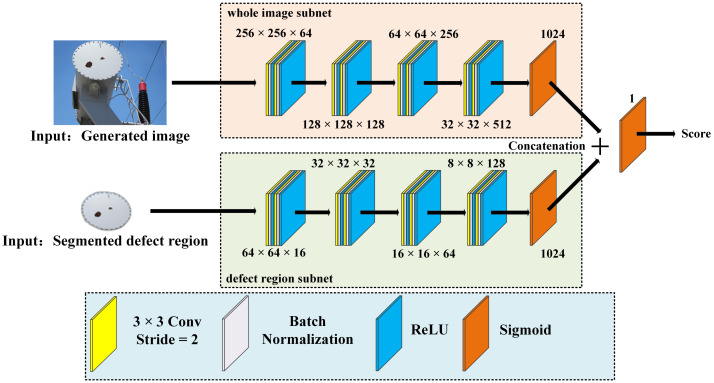
Joint discriminator for overall image and defect image.

The evaluation of generated images primarily focuses on two aspects. Firstly, it assesses whether the overall image accurately represents the characteristics of substation equipment. Secondly, it checks whether the generated defects align with the image features of oil leakage and rust defects. To address these criteria, this study proposes a joint discriminator for both the overall image and the defect image. This discriminator comprises two subnets: the defect region subnet and the whole image subnet. By constricting the image into feature maps, the discriminator combines the outputs of these subnets through a concatenation operation. This process judges the authenticity of the generated image, thus enhancing the quality of the generated defect. The diagram of the joint discriminator is illustrated in Figure 4.

As illustrated in Figure 4, the input of the overall image subnet is the generated entire image. This image is resized to 256×256 pixels and fed into the whole image subnet. It goes through 4 times of ConV-BN-ReLU-ConV-BN-ReLU operations and is finally processed by a Sigmoid layer, which outputs a 1-dimensional vector. Similarly, the defect region segmented by the mask is resized to 64×64 pixels and fed into the defect region subnet. It undergoes 4 rounds of ConV-BN-ReLU-ConV-BN-ReLU operations. ReLU is a linear function with output values in the range [0,∞). Its advantages include fast computation and a constant gradient in the positive interval, which avoids the vanishing gradient problem and allows the network to learn effective features more efficiently. However, its drawback is that the output is no longer restricted to the range [0,1], making it difficult to interpret as a probability. Therefore, after the ReLU function, we introduce the Sigmoid function. The output values of the Sigmoid function are in the range [0.5,1], allowing the output to be interpreted as probabilities, which is suitable for the scoring problem of the joint discriminator discussed in this paper. The magnitude of the score represents the probability of the image being authentic.

### 3.3. Loss Function

According to the structure of the ADD-GAN model, the designed loss function in this paper comprises three components: global perceptual loss, local defect perceptual loss, and cycle consistency loss [21]. The incorporation of the global perceptual loss in ADD-GAN facilitates the generation of defect images that possess both superior quality and diverse defects. The utilization of the local defect perceptual loss further enhances the detail authenticity of generated defects and semantic attributes. By incorporating the cycle consistency loss, ADD-GAN is able to generate defect images by learning image features from a limited collection of defective device images as well as a diverse range of normal device images.

#### 3.3.1. Global Perceptual Loss

Many GAN models frequently encounter mode collapse, resulting in the generation of poor-quality and less diverse images. This paper presents the utilization of the D2 adversarial loss [25] for improving the robustness of model training and enhancing the variability of features in the generated images. To account for the discriminator’s need to evaluate both the local imperfections and overall textures in the generated images, we have devised a locally perceptive D2 loss, building upon the foundation of the original D2 loss. By incorporating the improved global perceptual loss, we are able to enhance the quality of region-specific defects in the generated images. This enhancement allows for a more accurate assessment and refinement of the local imperfections, ultimately resulting in higher-quality generated images. For the generator $G_{norm2def}$, responsible for transforming defect-free images into defective images, and its corresponding discriminator $D1_{def}$, the loss function is formulated as follows:

(2) $$\begin{array}{c} L_{glo}\left(G_{norm2def};D1_{def};I_{def};MI_{def};F_{def};MF_{def}\right)=\\ E_{I_{norm}\in P_{data}\left(I_{norm}\right)}\left[lgD1_{def}\left(I_{def},MI_{def}\right)\right]\\ +E_{I_{def}\in P_{data}\left(I_{def}\right)}\left[lg\left(1-D1_{def}\left(F_{def},MF_{def}\right)\right)\right], \end{array}$$

where $I_{norm}$ represents the original defect-free image, $I_{def}$ represents the original device defect image, $MI_{def}$ represents the original defect image in mask area, $F_{def}$ represents the generated image, and $MF_{def}$ represents the generated image in the mask region. Equation (2) can be viewed as a process where the goal is to maximize the discriminator’s ability to detect local defects, represented by $D1_{def}$, while simultaneously minimizing the discrepancy between the generated image and its corresponding normal image in terms of local defect perception, represented by $G_{norm2def}$. The closer the value of $D1_{def}\left(I_{def},MI_{def}\right)$ approaches to 1, the more effectively the discriminator can differentiate the authentic sample. During the training phase, the discriminator is held fixed. If the generator $G_{norm2def}$ performs more effectively, the discriminator may make misjudgments, erroneously classifying generated images as original images. This increases the value of $D1_{def}\left(I_{def},MI_{def}\right)$, causing the latter part of Equation (2) to approach zero, thereby reducing the loss.

#### 3.3.2. Local Defect Perceptual Loss

Since surface defects on devices often exist only in a small portion of the surface, to further focus on the quality of generating defects, this paper introduces a local defect perceptual loss in the generator section of the network, as shown below:

(3) $$\begin{array}{c} L_{def}\left(MI_{norm};MF_{norm};MI_{def};MF_{def}\right)=L_{semantic}\left(MF_{norm};MI_{norm}\right)\\ +L_{semantic}\left(MF_{def};MI_{def}\right), \end{array}$$

where $L_{semantic}\left(MF_{norm};MI_{norm}\right)$ represents the difference loss between the defect-free portion of the image generated within the segmented mask and the real defect-free image, and $L_{semantic}\left(MF_{def};MI_{def}\right)$ represents the difference loss between the defective portion of the image generated within the segmented mask and the real defective image. The design purpose of this loss component is to ensure the quality of images in both the defective and defect-free regions within the mask during the training of the generator. A smaller loss indicates higher quality of images within the mask.

#### 3.3.3. Cycle Consistency Loss

In practical scenarios, the limited number of actual defect images compared to the abundant normal images may lead to a lack of diversity in the generated defect image features. This can be a challenge when using the local perceptual D2 loss to ensure both high quality and various characteristics in generated surface defect images. Therefore, this paper introduces cycle consistency loss [16] to prevent this situation and achieve the goal of generating defect images with rich features using normal images. The cycle consistency loss method assumes that a mapping $H_{def2norm}$ can transform the already generated defect images back into the space of defect-free sample images, thereby establishing a one-to-one mapping relationship between normal sample images and defect sample images spaces, avoiding the problem of monotonous features in generated defect sample images. The cycle consistency loss can be represented as follows:

(4) $$\begin{array}{c} L_{cyc}\left(G_{norm2def};H_{def2norm}\right)=E_{I_{norm}\in P_{data}\left(I_{norm}\right)}\left|\right|F_{norm}-I_{norm}\left|\right|\\ +E_{I_{def}\in P_{data}\left(I_{def}\right)}\left|\right|F_{def}-I_{def}\left|\right|. \end{array}$$

### 3.4. Optimizer

The training of a GAN model is a continuous game between the generator and discriminator models, involving the search for a Nash equilibrium point in a high-dimensional parameter space. This process is often unstable. The Adaptive Moment Estimation (Adam) [26] optimizer is an adaptive optimization algorithm that adjusts the learning rate based on historical gradient information. It combines ideas from both RMSProp and Momentum optimization algorithms, allowing adaptive adjustment of the learning rate for each parameter and normalizing the updates for parameters. This normalization ensures that each parameter update has a similar magnitude, thereby improving the model’s convergence speed and generalization ability, enhancing training effectiveness. The Adam optimizer performs exceptionally well in dealing with non-convex optimization problems such as GANs. Therefore, this paper utilizes the Adam optimizer in the training process of ADD-GAN.

Adam optimizer is a gradient descent algorithm that combines the momentum algorithm with an adaptive learning rate algorithm. It achieves faster convergence and better generalization by calculating an adaptive learning rate for each parameter. At each step, the Adam optimizer calculates the moving average of the gradients and the moving average of the squared gradients, using them to update the model parameters. Specifically, it uses two exponentially weighted moving averages to adjust the learning rate for each parameter, achieving the effect of an adaptive learning rate. The update rule is as follows:

(5) $$m_{t}=\beta_{1}\cdot m_{t-1}+\left(1-\beta_{1}\right)\cdot g_{t},$$

(6) $$v_{t}=\beta_{2}\cdot v_{t-1}+\left(1-\beta_{2}\right)\cdot g_{t}^{2},$$

(7) $$m_{t}^{\prime }=\frac{m_{t}}{1-\beta_{1}^{t}},$$

(8) $$v_{t}^{\prime }=\frac{v_{t}}{1-\beta_{2}^{t}},$$

(9) $$\theta_{t+1}=\theta_{t}-\frac{\eta }{\sqrt{v_{t}^{\prime }}+\epsilon }\cdot m_{t}^{\prime },$$

where $m_{t}$ is the moving average of the gradient at time *t*, $v_{t}$ is the moving average of the squared gradient at time *t*, $g_{t}$ is the gradient at time *t*, $\beta_{1}$ and $\beta_{2}$ are the exponential decay rates for the moment estimates, η is the learning rate, and ϵ is a small constant to prevent division by zero. This update rule adjusts the parameters based on both the first-order moment and the second-order moment of the gradients, providing adaptive learning rates for each parameter.

## 4. Experiments and Discussion

After constructing the ADD-GAN model, we conducted relevant experiments. The experiments were carried out using an IW4210-8G server with Ubuntu 18.04 as the operating system. Other configurations of the server are shown in Table 2. The ADD-GAN model was trained using GPU.

### 4.1. Dataset and Evaluation Metrics

In this paper, 700 images of substation equipment surface defects were collected through on-site scenarios, including 353 images of oil leakage on the surface and 347 images of metal rust. Additionally, 1200 images of normal equipment were collected. Subsequently, the paper employed a local defect image augmentation method. Firstly, 400 local defect images based on prior knowledge were manually segmented, including 200 images each for surface oil leakage and metal rust. Then, experts drew 400 local defect images based on their experience using tools, with 200 images each for surface oil leakage and metal rust.

These defect images obtained through local defect image enhancement were manually overlaid onto equipment images to generate new equipment defect images, forming the training dataset for ADD-GAN. The composition of the final training dataset is presented in Table 3.

In this paper, the defect image dataset generated by the ADD-GAN model is further input into three typical object detection models—YOLOv7-X, Faster R-CNN with VGG16 Net, and Single Shot MultiBox Detector (SSD)—for evaluation. The hyperparameters of these three models are shown in Table 4. Furthermore, several metrics, including mean average precision (mAP), precision, recall, and F1 Score, are used to assess the performance of the defect detection model. The specific calculation formulas are as follows:

(10) $$Precision=\frac{TP}{TP+FP},$$

where TP is True Positives, and FP is False Positives.

(11) $$Recall=\frac{TP}{TP+FN},$$

where FN is False Negatives.

(12) $$mAP=\frac{1}{n}\sum_{i=1}^{n}AP_{i},$$

where *n* is the number of classes, and $AP_{i}$ is the average precision for class *i*. $AP_{i}$ is the area under the precision–recall curve for class *i*.

(13) $$AP_{i}=\int_{0}^{1}P_{i}\left(R_{i}\right)dR_{i},$$

where $P_{i}\left(R_{i}\right)$ is the precision of class *i* under recall $R_{i}$.

(14) $$F1=\frac{2\cdot Precision\cdot Recall}{Precision+Recall},$$

### 4.2. The Effectiveness of Generated Data

**Figure 5:**
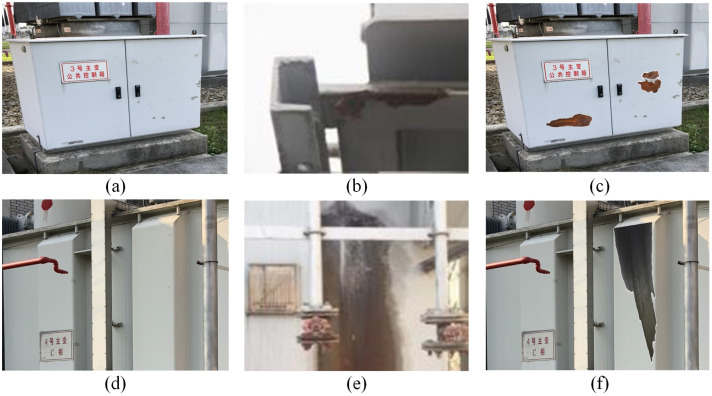
Defect image generated by ADD-GAN model: (**a**) Defect-free image. (**b**) The collected image of metal rust. (**c**) The generated rust image. (**d**) Defect-free image. (**e**) The collected image of surface oil leakage. (**f**) The generated surface oil leakage image.

**Figure 6:**
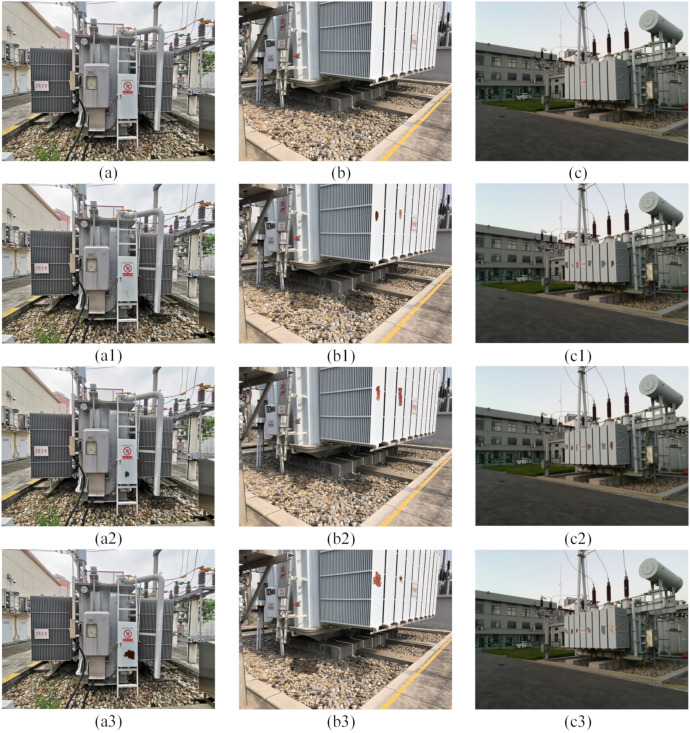
Generation results of ADD-GAN model: (**a**–**c**) Defect-free images. (**a1**–**c3**) The generated images.

After completing the construction of the dataset, we input defect-free images separately with two types of defect images, forming two training datasets, into the ADD-GAN model for training. After obtaining the trained generator model, defect-free images are input into the generator, and the generated images are shown in Figure 5 and Figure 6.

Figure 5a,d represent defect-free equipment images collected, Figure 5b shows the collected metal rust image of substation equipment, Figure 5e displays the oil leakage image obtained from collection, Figure 5c depicts the equipment image with rust defects generated by the ADD-GAN generator from Figure 5a, and Figure 5f illustrates the equipment image with oil leakage defects generated by the ADD-GAN generator from Figure 5d. Expert evaluation indicates that the generated equipment defect images exhibit a high degree of fidelity to real images and can be incorporated into training datasets for defect detection based on deep learning.

From Figure 6, it can be seen that the same image of a defect-free substation device can be used to generate images with different surface defects. This further demonstrates that the ADD-GAN model can effectively generate a large number of valid substation equipment surface defect images, thereby significantly expanding the defect image dataset.

In this study, using the ADD-GAN model, 1000 defect images of metal rust and oil leakage were generated and fused with the collected original defect images to form a defect dataset. This dataset was utilized to train three typical object detection models of YOLOV7, Faster R-CNN, and SSD. Performance comparisons were made with models trained using the original dataset, and the results are presented in Table 5. It is evident that incorporating the training set with generated data significantly improves the performance of the object detection models compared to the original dataset, thereby demonstrating the effectiveness of the ADD-GAN method.

### 4.3. Ablation Experiments

In order to validate the proposed local region defect generation network and the joint discriminator for overall image and defect image for improving the network’s performance, ablation experiments were conducted in this study. The ablation experiment involved commenting out the code for specific modules, thereby disabling related functionalities. This process led to the training of the corresponding generator network, generating a defect dataset. Subsequently, the generated defect dataset was utilized to create a defect image training set for training the YOLOv7 model. Finally, the trained detection model was evaluated using the same test set, and the results are presented in Table 6.

Analysis of Table 6 reveals that both the local region defect generation network and the joint discriminator contribute to the performance improvement of the ADD-GAN model. Through an examination of images generated by the relevant models in the ablation experiments, it can be observed that the local region defect generation network focuses more on the quality of generating local defects. It can segment local areas without altering overall image features, generating relevant defects within those local regions. The joint discriminator pays more attention to the overall image quality and the consistency of merging overall images with local defect images. It can rectify situations where there are clear boundaries between defect and normal areas in generated images, making the generated images more consistent with actual image features.

### 4.4. GAN Model Comparison Experiments

In order to further validate the image quality generated by the ADD-GAN model, this section designs comparative experiments. The first set of comparative experiments uses traditional image augmentation methods, such as image rotation, scale transformation, brightness transformation, etc., to generate a total of 2000 images of rust and oil leakage defects. These augmented images are then merged with the collected original defect images to create a dataset of equipment defects. The second to sixth sets of comparative experiments use baseline and improved methods [15,17,19], respectively, to generate 2000 defect images each. These generated images are combined with the collected original defect images to constitute datasets of equipment defects.

The complexity comparison of the six adversarial deep learning-based methods is shown in Table 7. This study compares the complexity of various models based on three metrics: Params, GPU Memory Usage, and total floating-point operations (GFLOPS).

It can be observed that the Defect-GAN model has the largest values for all three metrics: Params, GPU Memory Usage, and GFLOPS, indicating the highest complexity among the examined models. The complexity of the proposed ADD-GAN model and the Cycle-GAN model is on the same order of magnitude. From the perspective of the training set images, since the collected substation equipment images have a complex background, the other models transform and generate the overall style of the images. This leads to a higher degree of distortion in comparison to real-world scene images, posing challenges for image labeling and the training of the YOLOv7 model.

We compare the testing results of the YOLOv7 object detection models trained on datasets obtained by the various methods in real-world scene test sets. The results are shown in Table 7. From the analysis of the comparative experimental results, it can be concluded that the images generated by the ADD-GAN model are more diverse compared to those generated by traditional methods. Furthermore, compared to other generation methods like Cycle-GAN, the ADD-GAN model can control the region of defect generation, avoiding issues of realism caused by global style changes. The improved performance of typical object detection models further validates that the proposed ADD-GAN model is capable of generating equipment defect images with rich features and high realism.

### 4.5. Optimizer Comparison Experiments

**Figure 7:**
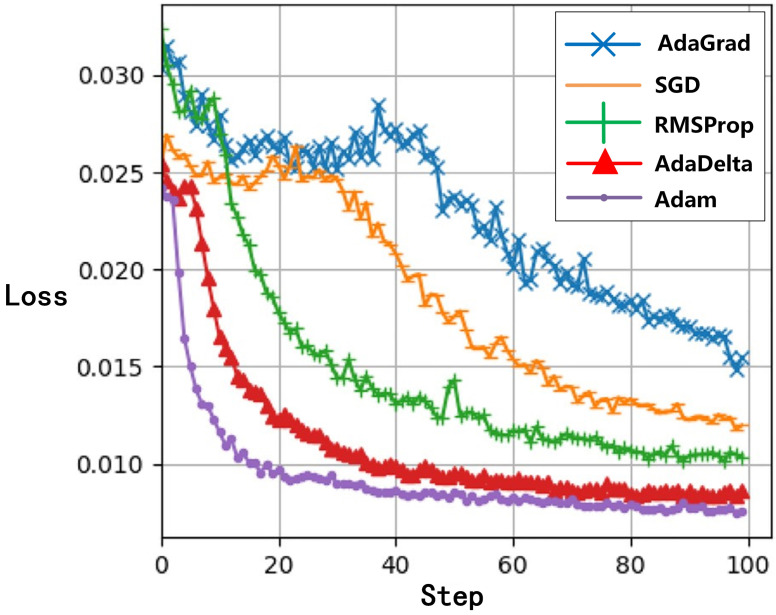
Comparison of loss curves for several optimizers.

In this paper, the Adam optimizer is employed in the training process of ADD-GAN. To validate the performance of the Adam optimizer, four commonly used optimizers—AdaGrad, RMSProp, SGD, and AdaDelta [27]—are used as a comparison in a designed experiment. Since the main purpose of optimizers is to improve the training process of neural networks, this paper compares the changes in the loss curves during the training process of the ADD-GAN model for various optimizers, as shown in Figure 7. It can be observed that the training process loss curve optimized by the Adam optimizer converges the fastest, with the smallest oscillation amplitude and the lowest final loss value. This is attributed to Adam combining momentum and adaptive learning rate algorithms in the gradient descent algorithm. It achieves faster convergence and better generalization by calculating the adaptive learning rate for each parameter.

**Figure 8:**
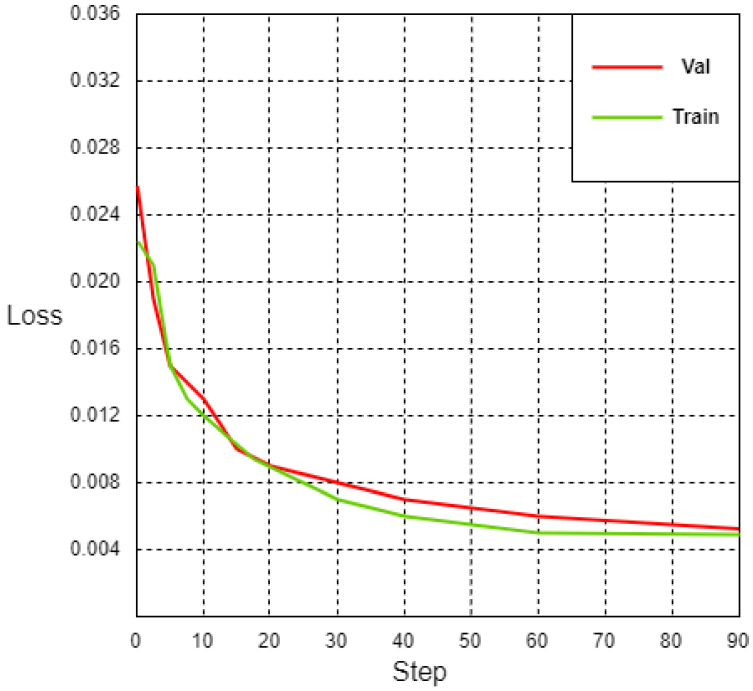
Comparison of train loss curve and validation loss curve for Adam optimizer.

To further validate the fitting performance of the ADD-GAN model trained with the Adam optimizer, we compared the training loss and validation loss, as shown in Figure 8. Both losses decrease and tend to stabilize, with a small gap between them. These results indicate that the ADD-GAN model trained with the Adam optimizer performs well on the training set and generalizes effectively to the validation set. This implies that the ADD-GAN model does not overfit or has a very low degree of overfitting.

The AdaGrad optimizer adjusts the learning rate by normalizing the gradient for each parameter. The normalization coefficient is calculated based on the sum of squares of all previous gradients. It can adaptively adjust the learning rate for each parameter but may hinder convergence due to the gradually accumulating gradient information leading to a too-small learning rate. SGD is one of the most basic and commonly used optimizers. It updates network parameters using the error of each sample, and each update only uses the gradient information of one sample, resulting in very fast computation. However, because it uses only one sample at a time, it may lead to oscillations or become stuck at local minima. The RMSProp optimizer calculates the second moment of the gradient information by weighted averaging to adaptively adjust the learning rate. It can adaptively adjust the learning rate, alleviating the issues in SGD. The AdaDelta optimizer is an improvement over AdaGrad. It calculates the second moment of the gradient information using a moving average and retains information only for a recent period. This optimizer can adaptively adjust the learning rate for each parameter and is not affected by the problem of continuously accumulating gradient information. However, in the model training process in this paper, the effects of these four optimizers are still not as good as the Adam optimizer.

### 4.6. Analysis of Joint Discriminator for Overall Image and Defect Image

The discriminator is a key component in GAN networks used to evaluate whether the generated images are real. A typical discriminator is a single-channel multi-layer convolutional network that extracts image features through earlier convolutional layers, and the final convolutional layer or fully connected layer is used to determine the authenticity of the image. Conventional discriminators only provide an overall assessment of the authenticity of an image and cannot focus on local details. Therefore, in the context of defect generation in this paper, the authenticity of generated defects may have limited impact on the overall score assigned to the generated images.

To address the mentioned issue and enhance the weight assigned to the authenticity of generated defects in the discriminator, this paper introduces a joint discriminator for the overall image and the defect image. This discriminator extends the global discriminator network by adding a defect region discriminator sub-network. This design aims to improve the quality of generated images with localized defects.

To validate the impact of the joint discriminator on image generation, this paper uses a discriminator composed of a global discriminator network as a comparison and trains the ADD-GAN model. In terms of complexity, the parameters of a single global discriminator network are 12.3 M, and the total floating-point operations are 38 GFLOPs. The parameters of the joint discriminator are 21.7 M, and the total floating-point operations are 71 GFLOPs. The joint discriminator has a higher complexity.

**Figure 9:**
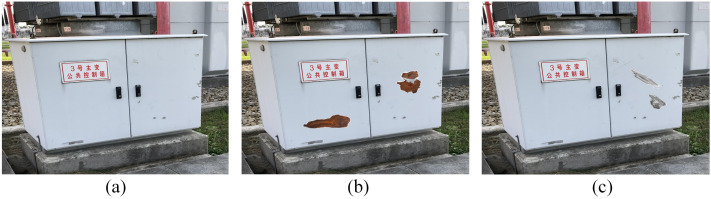
Comparison results of joint discriminator and global discriminator.

Regarding the image generation results, the generators trained with the two models are used to process normal images. The results are shown in Figure 9. Figure 9a depicts a normal device image, Figure 9b displays a defect image generated by the ADD-GAN network with the joint discriminator, and Figure 9c illustrates a defect image generated using only the global discriminator.

From the generated image results, it can be observed that the defect image generated by the discriminator without the addition of the local discriminator sub-network tends to preserve overall device characteristics rather than exhibiting rust defect features. This observation validates that a conventional discriminator tends to prioritize the overall authenticity of the generated image, potentially overlooking certain local defect image features.

In contrast, using the joint discriminator for overall image and the defect image not only preserves the overall authenticity of the image but also retains more local defect image features. This significantly enhances the defect image generation performance of the ADD-GAN model.

## 5. Conclusions

In this paper an ADD-GAN algorithm is proposed for generating rust and oil leakage defects on substation equipment images. Building upon adversarial deep learning, the algorithm introduces a local region defect generation network and a joint discriminator for the overall image and the defect image. This approach allows for the segmentation of local regions and generation of relevant defects without altering the global image features. Simultaneously, it pays attention to both the overall image quality and the fusion consistency between global and local defect images. As a result, ADD-GAN can generate high-quality equipment defect images with rich features and high realism. The experimental results demonstrate that the image quality generated by ADD-GAN surpasses traditional image augmentation methods and mainstream adversarial deep learning algorithms. Moreover, the training set generated by ADD-GAN effectively improves the detection accuracy of mainstream object detection algorithms.

Future work may involve refining the local region defect generation network for improved defect generation accuracy, conducting application experiments on a wider range of substation defect data, further validating the practical performance of ADD-GAN, and making additional enhancements to the network.

## Figures and Tables

**Table 1:** Guidelines during the defect drawing process.

	Surface Oil Leakage	Metal Rust
Color	Black, from light to heavy appears transparent to opaque	A mixture of brown, and yellow
Texture	Smooth, the edge part has some raised features	Rough, with a sense of granularity or hierarchy
Shape	There is no regular shape, and the edge is partially smooth	Irregular shape, rough edges
Distribution	A whole piece without fracture	Irregular
Reflection of light	Yes	No

**Table 2:** IW4210-8G server configuration.

Configuration	Parameters
CPU	Intel(R) Xeon(R) Silver 4214 CPU @ 2.20 GHz
CPU MHz	1000
CPU Cache	16,896 KB
RAM	257,580 GB
GPU	NVIDIA GeForce RTX 2080 Ti
VRAM	11 GB GDDR 6
Graphics Memory	14,000 MHz
Core	1350–1545 MHz

**Table 3:** Composition of the training set.

Image Categories	Image Source	Number	Total
Defect-free	On-site acquisition	1200	1200
Surface oil leakage	On-site acquisition	353	
	Manual segmentation	200	753
	Expert drawing	200	
Metal rust	On-site acquisition	347	
	Manual segmentation	200	747
	Expert drawing	200	

**Table 4:** Hyperparameters of three typical object detection models.

Hyperparameters of Defect Detection Model	YOLOv7-X	Faster R-CNN with VGG16 Net	SSD
Parameters/MB	45.3	154.7	27.4
GFLOPS	151	375	97
Input image size	640 × 640	500 × 500	300 × 300
Initial learning rate	0.01	0.001	0.01
Final learning rate	0.1	0.01	0.1
Momentum	0.937	0.9	0.9
Weight decay	0.0005	0.0001	0.0005
Batch size	8	32	64

**Table 5:** The performance comparison experiment.

The Types of Training Datasets	Detection Model	mAP/%	Precision/%	Recall/%	F1 Score /%
Original images	SSD	68.3	75.2	74.4	74.8
	Faster R-CNN	70.2	77.8	77.1	77.4
	YOLOv7	71.9	81.4	80.8	81.1
Original images + generated images	SSD	**74.1**	**81.2**	**80.8**	**81.0**
	Faster R-CNN	**75.3**	**84.9**	**84.3**	**84.6**
	YOLOv7	**81.5**	**90.3**	**87.2**	**88.7**

**Table 6:** Ablation experiment results.

Detection Model	mAP/%	Precision/%	Recall/%	F1 Score /%
Without the local region defect generation network	78.3	86.9	82.7	84.7
Without the joint discriminator	79.5	87.2	84.5	85.8
ADD-GAN	**81.5**	**90.3**	**87.2**	**88.7**

**Table 7:** Comparison experiment results.

Image Augmentation Method	mAP/%	F1 Score /%	Params/MB	GPU Memory Usage/G	GFLOPs
Traditional methods	75.8	85.3	-	-	-
Cycle-GAN	74.5	85.1	40.5	6.4	112
U-ResNet + Cycle-GAN	74.8	85.0	42.4	6.4	136
DCGAN	72.6	81.9	28.7	4.8	87
USCONV+DCGAN	73.2	83.6	28.9	4.8	94
Defect-GAN	77.4	87.3	**77.4**	**11.3**	**357**
ADD-GAN	**81.5**	**88.7**	45.3	7.1	151

## Data Availability

Data are contained within the article.
